# Supplementary material for: Trends and socioeconomic inequalities of recommended antenatal care services utilization in Ethiopia: A decomposition analysis using Ethiopian nationwide Demographic Health Surveys 2011–2019
Source: PLoS One. 2025 Feb 4;20(2):e0318337. doi: 10.1371/journal.pone.0318337 (PMC11793738; doi:10.1371/journal.pone.0318337)
Supplement: Attachment — Submitted filename: Rebutal letter with point by point response.docx [file pone.0318337.s002.docx]

Date: October 16, 2024

**To: PLOS ONE**

**Subject: Submission of Revised Manuscript**

Dear Editor and Reviewer,

We, the authors, are deeply appreciative of your response to our manuscript **# PONE-D-24-13694**, titled **"Trends and Socioeconomic Inequality in Recommended Antenatal Care Service Utilization in Ethiopia: A Decomposition Analysis Using Ethiopian Nationwide Demographic Health Surveys Data from 2011-2019**". Your valuable comments, and expert suggestions are instrumental to enhance the quality of our work.

We have not used retracted references and have meticulously revised and updated the manuscript taking the editor and reviewer’s comments and suggestions into consideration. We are eager to publish the manuscript in your reputable journal to reach a relevant audience and influence policy changes aimed at improving antenatal care services in Ethiopia and similar contexts.

Once again, we are grateful for your comments and suggestions.

Best regards,

Corresponding author: Yawkal Tsega

On behalf of the authors,

Email: [yawkaltsega@gmail.com](mailto:yawkaltsega@gmail.com)

Mobile: +251933559351

**Response to Editors comment**

**Comment 1:** Please note that we have only been able to secure a single reviewer to assess your manuscript. We are issuing a decision on your manuscript at this point to prevent further delays in the evaluation of your manuscript. Please be aware that the editor who handles your revised manuscript might find it necessary to invite additional reviewers to assess this work once the revised manuscript is submitted. However, we will aim to proceed on the basis of this single review if possible.

**Authors’ response**: Dear Editor, thank you for your update regarding the review process. We appreciate the efforts made to secure a reviewer for our manuscript. We revised the manuscript and proofread for grammatical errors and submitted the revised manuscript with rebuttal letter and point by point responses.

**Response to Reviewer 1 comments**

**Comment 1:** This manuscript analyzed the patterns and socioeconomic disparities in the utilization of recommended antenatal care (ANC) services in Ethiopia. It used nationwide demographic health survey data from 2011 to 2019. In general, the research is solid, carefully designed, and the paper is skillfully written and well-structured. Having said that, I strongly urge the authors to focus on the following few issues:

**Authors’ response**: Dear reviewer 1, thank you for reviewing our manuscript and your encouraging kind words. We addressed all the comments, suggestions and recommendations in the revised manuscript as per your comments and suggestions.

**Comment 2:** Some attention is needed for lines 207-208 by the authors.

**Authors’ response**: Thank you for your comments. We addressed the comment in the revised manuscript.

**Comment 3:** Line 232: The authors wrote: (Error! Reference source not found.). What is the meaning of this statement?

**Authors’ response**: Thank you for bringing this editorial error into our attention. We revised statement in the revised version of the manuscript. It was due to the error of Table citation.

**Comment 4:** Unlike the Tables, all Figures clearly require significant revisions to enhance their quality.

**Authors’ response**: We are grateful for your comments and suggestions. We tried to increase the quality of figures to increase the visibility.

**Comment 5:** Editing is needed for the cell in Table 6, specifically in column 3 and row 4.

**Authors’ response**: Thank you for your suggestion. We revised and proofread table 6 and the overall contents of the manuscript.

**Comment 6:** While the discussion is acceptable, it could be enhanced by including more comparisons with other studies conducted in Ethiopia or the surrounding region that share similar conditions.

**Authors’ response**: Thank you for your valuable feedback. We appreciate the suggestion to include more comparisons with other studies conducted in Ethiopia or the surrounding region. We incorporated relevant studies to enhance the discussion and provide a more comprehensive analysis of the conditions and findings.
