# Supplementary material for: Trends and socioeconomic inequalities of recommended antenatal care services utilization in Ethiopia: A decomposition analysis using Ethiopian nationwide Demographic Health Surveys 2011–2019
Source: PLoS One. 2025 Feb 4;20(2):e0318337. doi: 10.1371/journal.pone.0318337 (PMC11793738; doi:10.1371/journal.pone.0318337)
Supplement: Attachment — Submitted filename: Rebutal letter.docx [file pone.0318337.s003.docx]

Date: 08 December 2024

**To: PLOS ONE**

**Subject: Submission of Revised Manuscript**

Dear Editor and Reviewers,

We, the authors, are deeply appreciative of your response to our manuscript **# PONE-D-24-13694R1**, titled **"** **Trends and socioeconomic inequalities of recommended antenatal care services utilization in Ethiopia: A Decomposition analysis using Ethiopian nationwide Demographic Health Surveys 2011-2019**". Your valuable comments, and expert suggestions are instrumental to enhance the quality of our manuscript.

We have revised the manuscript taking the reviewers’ comments and suggestions into consideration. We are eager to publish the manuscript in your reputable journal to reach a relevant audience and influence policy changes aimed at improving antenatal care services in Ethiopia and other similar contexts.

Once again, we are grateful for the editor and reviewers for expert comments, recommendations, and suggestions.

Best regards,

Yawkal Tsega

On behalf of the authors

**Response to Editors comment**

**Comment 1:** Thank you for submitting your manuscript to PLOS ONE. After careful consideration, we feel that it has merit but does not fully meet PLOS ONE’s publication criteria as it currently stands. Therefore, we invite you to submit a revised version of the manuscript that addresses the points raised during the review process.

**Authors’ response**: Dear Editor, Thank you so much for your update and time in evaluating our manuscript. We revised and tried to address all the comments and suggestions provided by the reviewers and submitted the revised manuscript.

**Response to Reviewer 2 comments**

**Comment 1:** In the methodology, the authors have kept individual level socioeconomic status and community or macro indicators such as HDI together, it will be good if they present it with separate subtitle.

**Authors’ response**: Dear Reviewer 2, we are grateful for your insightful comments and suggestions. Your comments and suggestions have been instrumental in enhancing the quality of our work. Based on your feedback, we have separated the individual and community-level factors in the revised manuscript.

**Comment 2:** Further, Mother’s current marital status is described as currently unmarried, currently married. It will be good if currently unmarried presented as widowed/separated

**Authors’ response**: Thank you for your insightful recommendations. We have clarified the meaning of "currently unmarried" (single/divorced/widowed) in the footnote of the table in the revised manuscript, taking your suggestions into consideration.

**Comment 3:** In table 3, for the Family size, in the COR the authors have kept ≥7 as reference and in the adjusted model, the authors have kept 1-4 as reference, why? It’s better to follow same

**Authors’ response**: Thank you so much for detailed comments of the manuscript. It was due to editorial errors. We have corrected it in the revised manuscript.

**Response to Reviewer 3 comments**

**Comment 1:** Overview: This paper, submitted to PLOS ONE, has focused on an important public health issue in LICs, socioeconomic inequality in utilization of antenatal care services among women. The study used data from three waves of Ethiopian DHS conducted in years 2011, 2016, and 2019. Bivariate and multivariate logistic regression models were applied to find out the determinants of recommended ANC services utilization among Ethiopian mothers. Further, decomposition of concentration index was carried out to measure contributions of studied variables in socioeconomic inequalities.

Finding highlights that coverage of recommended ANC services increased from 29.96% in 2011 to 44.70% in 2019. Several socio-demographic factors such as mother’s age and education, household wealth status, distance of mothers’, place to the nearest health facility, among others were significantly associated with utilization of recommended ANC services. The decomposition of concentration indices revealed household wealth status as the largest contributor (~67%) to the socioeconomic inequality that exist in utilization of antenatal care services among Ethiopian women.

**Authors’ response**: Dear reviewer 3, we appreciate for your time, expert comments, suggestions, and recommendations. Your detailed comments have played significant role in the quality improvement of our work. We have tried to address all your suggestions, and comments in the revised manuscript.

**Comment 2:** The current version of the paper does not meet the publication quality. The manuscript should be thoroughly revised by carefully addressing a number of shortcomings, ranging from its grammatical errors to statistical analyses. Authors may consider my comments below while revising the manuscript. I appreciate the opportunity to review this work and hope that authors would find my comments useful.

**Authors’ response**: Your comments are highly valuable for the quality of our work, and we appreciate them. We have repeatedly proofread the manuscript and addressed the grammatical errors, punctuation errors, and logical flow.

**Comment 3:** The information presented in the ‘Introduction’ section is very generic. The research problem to be addressed (in this study) should be described adequately and with an appropriate background, which is missing.

**Authors’ response**: Thank you for your deep and detailed comments. We have revised the introduction section to better explain the research problems, existing literature, and the gap this research fills, taking your suggestions into consideration.

**Comment 4:** Where is the literature review? Authors should add at least one paragraph on review of relevant literature to the ‘Introduction’ section.

**Authors’ response**: We are grateful for your recommendation. We added literatures in the revised manuscript tried to improve the introduction part in the revised manuscript.

**Comment 5:** Next is research gap. Authors should describe whether there were any research gaps in the existing literature and thereafter, the need for this study should be mentioned.

**Authors’ response**: We have thoroughly revised the introduction section in the revised manuscript. We have explained the rationale for the current study by highlighting the gaps in the existing literature.

**Comment 6:** The methodology used in the study should be introduced to the readers in the ‘Introduction’ section only.

**Authors’ response**: We summarized the methods used in the study in the revised manuscript.

**Comment 7:** Line 80: Authors may consider renaming of the heading ‘Data and Methods’.

**Authors’ response**: We are grateful for your expert comments and suggestions. We clearly wrote the headings according to PLOS ONE manuscript submission guideline.

**Comment 8:** Line 89: What is ‘source population’?

**Authors’ response**: Dear Reviewer 3, we are grateful for your expert comments and suggestions. We clearly stated the source and study populations in the revised manuscript. *“All pregnant women five years preceding each respective survey year were the source populations, and all pregnant women five years preceding each survey year in the selected EAs were the study populations”.*

**Comment 9:** Line 90-91: It should be mentioned clearly about who were the study population (e.g., pregnant women or women of reproductive age).

**Authors’ response**: We appreciate your expert comments and suggestions. We clearly stated the source and study populations in the revised manuscript. *“All pregnant women five years preceding each respective survey year were the source populations, and all pregnant women five years preceding each survey year in the selected EAs were the study populations”.*

**Comment 10:** How do you define ‘Recommended ANC Services’? Please disclose it to readers while defining outcome variables.

**Authors’ response**: Thank you so much. Recommended ANC services utilization was considered *“if the mother attended her first ANC visit within first trimester of her pregnancy and if she completed at least four ANC visits”.* We operationalized the outcome variable, recommended ANC services utilization in the revised manuscript.

**Comment 11:** Line 115: Remove the statement “and it is also more practical to implement”

**Authors’ response**: We revised the statement in the revised manuscript as per your suggestions.

**Comment 12:** Line 199-201, 202-203: Not clear, what authors want to state.

**Authors’ response**: From line 199-203, we stated the ethical approval. We revised the section and tried to make it clearer in the revised manuscript.

**Comment 13:** Line 218: What does it mean by ‘candidates’?

**Authors’ response**: Thank you so much for your question. We revised the statement in the revised manuscript. It was just to explain the eligible variables for multivariable logistic regression.

**Comment 14:** Line 401-403: Not clear, what authors want to state.

**Authors’ response**: We greatly appreciate your insightful and expert comments. In lines 401-403, we intended to explain the policy implications of the study findings. However, we have revised the paragraph to be more specific and clearer in the revised manuscript, taking your suggestions into consideration.

**Comment 15:** The study should be organized appropriately ensuring that it is not taxing the readers’ attention.

**Authors’ response**: We conducted a thorough proofread of the whole manuscript word by word and tried to enhance the organization and quality of the manuscript in the current revised manuscript.

**Comment 16:** Finally, this manuscript needs thorough English language editing to make it understandable to the readers.

**Authors’ response**: Dear Reviewer 3, we are very grateful for your insightful and expert comments, suggestions, and recommendations. We have gladly accepted your suggestions and conducted a thorough proofread of the entire manuscript, word by word, to enhance its organization and quality in the revised version.
